# On-Chip Integration of a Plasmonic FET Source and a Nano-Patch Antenna for Efficient Terahertz Wave Radiation

**DOI:** 10.3390/nano13243114

**Published:** 2023-12-11

**Authors:** Justin Crabb, Xavier Cantos-Roman, Gregory Aizin, Josep Miquel Jornet

**Affiliations:** 1 Department of Electrical and Computer Engineering, Northeastern University, Boston, MA 02115, USA; cantosroman.x@northeastern.edu; 2 Kingsborough College, The City University of New York, Brooklyn, NY 11235, USA; gregory.aizin@kbcc.cuny.edu

**Keywords:** graphene, transistor, plasmonic, terahertz, antenna

## Abstract

Graphene-based Field-Effect Transistors (FETs) integrated with microstrip patch antennas offer a promising approach for terahertz signal radiation. In this study, a dual-stage simulation methodology is employed to comprehensively investigate the device’s performance. The initial stage, executed in MATLAB, delves into charge transport dynamics within a FET under asymmetric boundary conditions, employing hydrodynamic equations for electron transport in the graphene channel. Electromagnetic field interactions are modeled via Finite-Difference Time-Domain (FDTD) techniques. The second stage, conducted in COMSOL Multiphysics, focuses on the microstrip patch antenna’s radiative characteristics. Notably, analysis of the S11 curve reveals minimal reflections at the FET’s resonant frequency of 1.34672 THz, indicating efficient impedance matching. Examination of the radiation pattern demonstrates the antenna’s favorable directional properties. This research underscores the potential of graphene-based FETs for terahertz applications, offering tunable impedance matching and high radiation efficiency for future terahertz devices.

## 1. Introduction

The recent years of escalating demands for faster internet connectivity, driven by the increase in online applications and number of users [1], sparked the imperative quest for wireless data rates in the terabit per second range. Such ambitious rates require a leverage of higher bandwidths available in the terahertz (THz) band (0.1 to 10 THz) of the electromagnetic (EM) spectrum [2]. The terahertz domain holds promise for various applications, such as the realm of short-range wireless connectivity. For instance, terahertz time-domain spectroscopy (THz-TDS) [3] offers many opportunities ranging from noninvasive imaging [4] to biomedical science [5]. Additionally, THz-TDS enhances the study of charge carrier transport in materials such as semiconductors [6] and graphene [7,8]. Advances in Wireless networks on chip (WNoC) [9] have led to opportunities for on-chip and chip-to-chip communication, addressing wiring delays and enhancing the compactness of input/output (I/O) ports. Concurrently, the deployment of wireless nanosensor networks [10], comprising chemical sensors, biosensors, and physical sensors, alongside their integration into macro-scale networks within the Internet of Nano-Things [11], is anticipated to revolutionize industries such as healthcare, biomedicine, and defense. However, the realization of these transformative applications is impeded by the absence of a compact, efficient, and room-temperature operating terahertz source for high-power, low-noise radiation.

A promising approach toward developing an active terahertz source involves utilizing electron plasma oscillations in the two-dimensional (2D) electron channel of field-effect transistors (FETs). Many 2D materials have been proposed for terahertz applications due to their appealing electronic and optical properties, including a tunable bandgap, high carrier mobility, and wideband optical absorption [12]. Recent experiments with graphene FETs have demonstrated high-quality 2D electron plasma oscillations in the terahertz domain at room temperatures [13,14]. The inherent advantages of FETs, such as their compact size (typically a few microns), compatibility with planar CMOS technology and their ease of external control over plasma amplitude and frequency, position them as highly attractive candidates for the development of tunable terahertz sources. Our previous works involve the development of the multiphysics simulation platform of a graphene-based plasmonic terahertz generator [15], designing the FET based on experimental setups [16], and integrating on-chip modulation to the FET [17].

While plasmonic FETs offer a compact package for terahertz signal generation, EM radiation often lacks gain and directivity. To combat this issue, several passive elements can be applied to the devices to help bridge the terahertz gap, such as graphennas [18,19,20]. Graphene supports the propagation of tightly confined Surface Plasmon Polariton (SPP) waves with a reduction in propagation speed in comparison to its vacuum EM propagation speed counterpart. These graphene-based plasmonic nano-antennas take advantage of the high confinement factor in graphene, reducing the antenna size to a few μm, up to two orders of magnitude below the size of metallic antennas in the terahertz regime [21]. The radiation pattern of a graphenna is similar to an equivalent metallic antenna, with comparable efficiencies. This compact setup and ease of integration fit well in WNoCs and other nanosystem applications. However, for longer propagation distances, a larger radiating element is required, such as a metallic antenna.

In addition to graphennas, lenses may be applied to directional antenna and graphenna systems to further enhance their radiation efficiency, or applied directly to the source. Terahertz lenses can be implemented in several ways, such as fixed, typically with dielectric materials [22,23], or programmable, typically with metamaterials [24,25], coming in a hemispherical shape of a few hundred microns to a few hundred millimeters in size. Their simplicity allows seamless integration into several terahertz systems, such as waveguides, horn antennas, antenna array systems, on-chip antennas, and FETs, to name a few.

Traditional metallic antennas for passive terahertz emission, reviewed in [26], include horn antennas [27], dipole antennas, and microstrip antennas [28], and provide a larger output power due to their large size. Horn antennas offer a highly directive, rotationally symmetric beam pattern with a gain above 30 dBi, low cross-polarization level of −30 dB with 98% coupling efficiency [29,30]. Microstrip antennas, substrate-based planar antennas, come in a variety of forms such as butterfly, U-shaped, log-periodic, and patch antennas. While microstrip antennas typically exhibit a lower gain compared to horn antennas [31], this trade-off is offset by their inherent advantages, including a compact form factor, ease and low cost of fabrication, and higher degree of tunability, enabling precise impedance matching and optimization of their radiative performance.

In this study, we design a graphene FET terahertz source integrated with a microstrip patch antenna, facilitated through a tailored metamaterial feed structure. A microstrip patch antenna is chosen for its ease of implementation and integration into the FET system without destroying the asymmetric boundary conditions required for our device setup. Leveraging our computational framework, which self-consistently solves electron transport and electromagnetic equations in MATLAB [15] with antenna engineering simulations in COMSOL, we aim to optimize the radiation characteristics of the FET–antenna system. Graphene-based FET is constructed based on our previously developed model [16]. It features the asymmetric geometry [32] designed to facilitate the boundary conditions required for the implementation of the Dyakonov–Shur (DS) instability in the transistor. This instability induces the onset of plasmonic oscillations in the FET channel within the terahertz frequency band. To enable efficient coupling from the plasmonic channel to the gate and subsequently sufficient current flow to the antenna via the feed, we employ a Hyperbolic Metamaterial (HMM) in the gate/feed system. Our results show that the efficient coupling of the plasmons in graphene to the gate along with the proposed antenna and feeding system should enhance the radiation from the graphene FET by nearly 20 times.

In contrast, we highlight relevant studies demonstrating on-chip terahertz generation integrated with antenna structures. Notably, Tong et al. [33] present a graphene-based FET coupled to a double-patch antenna and an on-chip silicon lens, both numerically and experimentally. This configuration yields an output power on the order of a few nW, with the double patch also serving as the source and drain contacts for the FET. Operating within the 1–3 THz range at an optimal peak at 2.1 THz, the device functions at room temperature and offers versatility as both an emitter and a detector. On the spectrum of on-chip implementations of antennas, various approaches [34,35,36,37,38,39] employ frequency multiplying chains on chip, requiring an external RF source. These designs typically operate below 1 THz, achieving power outputs upwards of 20 dBm. Additionally, the photoconductive antenna method [40,41,42,43,44,45] entails an arrangement requiring an external optical source. In this setup, a photoconductive semiconductor, subjected to an applied external field, is irradiated by the optical source, emitting pulses in the femtosecond range, ranging from 0.1 to a few THz, with power outputs reaching a few mW.

Recent developments in on-chip terahertz sources without external antennas include Quantum Cascade Lasers (QCLs), High Electron Mobility Transistors (HEMTs), and Resonant Tunneling Diodes (RTDs) [46]. QCLs, relying on optical transitions in repeated stacks of semiconductor nanostructures [47,48,49], have seen substantial progress, achieving tunability within 1–5 THz and emitting over 1 W in pulsed mode at 10 K [50]. However, challenges persist in reaching room temperature operation conditions while generating signals in the continuous wave regime. HEMTs employ various heterojunctions of semiconductors and rely on a plasmonic 2D channel to offer compact terahertz sources at room temperature capable of breaching the 1 THz limit [51,52]. However, these plasmonic devices are relatively weak, emitting few μW of power [53], and the device physics are often a design challenge. On the other hand, RTDs are constructed with ultrathin layers of a large-bandgap semiconductor within a lower-bandgap semiconductor [54,55]. While recent developments have shown a considerable increase in fundamental operation frequency reaching 1.98 THz [56], designers face challenges related to limited output power (typically on a sub-mW level), limited frequency range, and parasitic capacitances. The integration of RTDs into power combining arrays has enhanced power output, reaching 0.73 mW at 1 THz [55,57].

## 2. Materials and Methods

The proposed device amalgamates a microstrip patch antenna with a FET featuring a graphene channel. This union is achieved through a meticulously engineered feed structure, with the key importance of not breaking the asymmetric boundary conditions of DS instability. The device architecture encompasses several integral components, each designed to facilitate active, efficient terahertz signal radiation. This section breaks down the system into several components to present the intricacies of the device geometry.

### 2.1. Field-Effect Transistor

**Figure 1:**
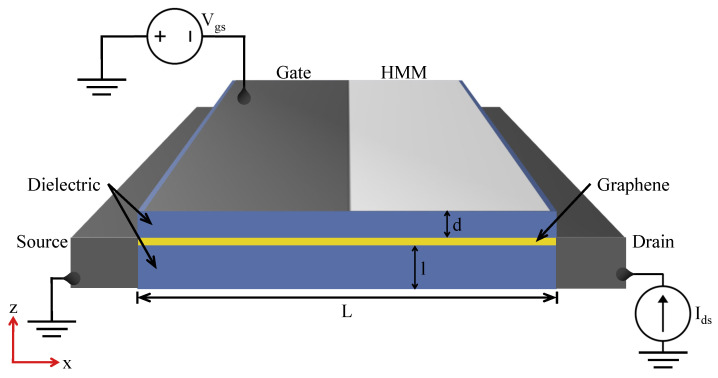
Schematic of the graphene-based FET with the applied gate voltage and drain current; the plasmonic current within the graphene layer coupled to the gate is carried to the feed through the HMM. The channel is asymmetrically depleted via the bias voltage.

At the core of our device lies the FET, displayed in Figure 1, a critical component in the system in charge of generating the terahertz signal. The graphene layer serves as the plasmonic cavity with a channel length (*L*) of 1 μm, sandwiched between two silicon dioxide (SiO${}_{2}$) dielectric layers serving as the top barrier and bottom substrate, with depths of *d* = 35 nm and *l* = 4.95 μm, respectively. A metallic reflector is placed below the substrate to confine the EM waves within the device. The gate contact is asymmetrically positioned atop the dielectric barrier closer to the source contact, giving rise to an asymmetrical configuration. Superimposed upon the upper SiO${}_{2}$ layer to the right of the metallic gate is the HMM—a pivotal element in our device’s functionality.

The physical mechanism to convert the plasma oscillations in the FET electron channel into an EM signal was proposed by Dyakonov and Shur in ref. [58]. The authors theoretically demonstrated that in a FET biased by a DC current with a plasmonic cavity formed in the FET channel, the plasma wave amplitude may exponentially increase after multiple reflections from the source and the drain contacts. This instability occurs if asymmetric boundary conditions are imposed at opposite ends of the plasmonic cavity for plasma wave reflections, specifically in the case of a large AC impedance between the ohmic contact and the gate at one side of the FET cavity (the drain side, $Z_{gd}$) and a small AC impedance at the opposite end (the source side, $Z_{gs}$) when a DC current flows from the drain to the source. In the instability endpoint, stationary sustained plasma oscillations are developed in the plasmonic channel, and the energy supplied by the DC current is balanced by Joule losses and radiation [15,59,60].

In early works, ideal boundary conditions with gate–source impedance $Z_{gs}=0$ and gate–drain impedance $Z_{gd}=\infty$ were assumed [58]. It was later shown that DS instability takes place at any finite boundary impedance provided that $Z_{gd}>Z_{gs}$ and Joule losses are sufficiently small [16,61]. Practical implementations of the finite asymmetric boundary impedances rely on geometric asymmetry in the FET. Previous experimental demonstrations were exploited by shorting the source and gate contacts [51], asymmetrically positioning the gate contact with respect to the source and drain contacts [62], depleting the channel on one side, and an engineered FET structural asymmetry [32]. Consequently, resonant terahertz emission at plasma frequencies tunable by the gate voltage was observed. Transport measurements in these structures demonstrated sustainable plasma oscillations in the FET cavity consistent with DS instability. Our simulated FET utilizes a combination of asymmetries to realize DS instability in the plasmonic cavity for terahertz signal generation, including applied gate voltage $V_{gs}$ asymmetrically depleting the channel, with applied current $I_{ds}$ ensuring electron flow for the plasmonic cavity.

### 2.2. Hyperbolic Metamaterial

The employment of the HMM as a pivotal element in our device architecture is substantiated by its anisotropic behavior suitable for our objective. A specific placement, geometry, and design is required to match the HMM to the system without destroying the DS boundary conditions. The dispersion of the HMM conforms to a hyperboloid shape [63], with one directional component of permittivity (ε) extending infinitely in the direction where ε is negative. Effective metamaterials with hyperbolic dispersion have been experimentally realized using layered metal–dielectric structures in a repeated stack, or via nanowire arrays, in the terahertz regime [64], generally achieved using a semiconductor such as graphene as opposed to a metal in the layered structure [65], and also in the optical range [66], for sub-wavelength imaging [67,68], focusing [69], and other applications [70,71,72]. A common approach is to use highly conductive metals, such as gold or silver, for the metallic component, and dielectrics such as SiO_2_ for the dielectric component. We opt to use a gold-SiO_2_ stacked layer design in our approach.

Notably, the HMM plays a dual role, functioning as both a dielectric in the out-of-plane direction and a metal in the in-plane direction. Specifically, the properties of the HMM are harnessed to behave as a dielectric in the direction where the permittivity (ε) is negative, while concurrently behaving as a metal in the orthogonal direction [73]. This utilization of the HMM should endow us with the sought-after asymmetry in gate–source and gate–drain capacitances and impedances. We note that the asymmetry is only in the *z* direction, but our numerical simulations in this work demonstrate this is sufficient for the onset of DS instability. The choice of depths (thickness of the stacked layers) also influences the HMM’s behavior, with thinner layers favoring metallicity and thicker layers favoring dielectric behavior. Thinner layers are used in our simulation to efficiently carry the current through the gate to the feed while still providing enough asymmetry in the z-direction for the onset of DS instability. In this direction where the HMM layer serves as a dielectric, minor reflections are experienced due to the discontinuity in permittivities of the top dielectric barrier and the HMM. These minor reflections contribute to the constructive interference of terahertz waves within the FET mentioned in the previous section, enhancing the device’s overall performance by further confining the EM radiation within the device.

Furthermore, the regions of the dispersion relation that extend to infinity signify the existence of extremely large wavevectors (*k*) or infinitesimally short wavelengths (λ) that satisfy the dispersion relation equation for the HMM given by $\left(\kappa_{x}^{2}+\kappa_{y}^{2}\right)/\epsilon_{zz}+\left(k_{z}^{2}\right)/\epsilon_{xx}=\omega^{2}/c^{2}$ [74]. This, in turn, implies the presence of an unbounded photonic density of states within the material, rendering it exceptionally efficient for both radiation and absorption processes. Thus, the plasmonic oscillations in the channel should efficiently couple to the metallic gate and the HMM. The efficiency of this coupling is rooted in the unique properties of graphene, specifically the plasmonic waves generated by DS instability, characterized by strong electron density oscillations known to interact strongly with nearby structures. The infinite photonic density of states in the HMM facilitates efficient plasmonic coupling by enabling an abundance of available electromagnetic states, allowing for increased interactions and efficient transfer of plasmonic energy to the material. In our device configuration, the HMM assumes the role of a metal type (type II, $\varepsilon_{⊥}<0$ and $\varepsilon_{\|}>0$) on the right side of the gate, aligned parallel to the two-dimensional electron gas (2DEG), while acting as a dielectric in a perpendicular dimension (*z*). The effective dielectric tensor components for the parallel $\varepsilon_{\|}$ and perpendicular $\varepsilon_{⊥}$ direction with respect to the anisotropy axis is given by [75]

(1) $$\varepsilon_{⊥}=\frac{\varepsilon_{m}d_{m}+\varepsilon_{d}d_{d}}{d_{m}+d_{d}},\frac{1}{\varepsilon_{\|}}=\frac{\frac{d_{m}}{\varepsilon_{m}}+\frac{d_{d}}{\varepsilon_{d}}}{d_{m}+d_{d}},$$

where $d_{m}$ ($d_{d}$) is the thickness and $\varepsilon_{m}$ ($\varepsilon_{d}$) is the dielectric constant of the metallic (dielectric) component. By tuning these parameters such that $\varepsilon_{\|}\varepsilon_{⊥}<0$, we can attain the hyperbolic regime. This type II configuration facilitates the controlled flow of current into the antenna.

### 2.3. Microstrip Patch Antenna and Feed

The microstrip patch antenna, a vital component for efficient terahertz signal radiation, is meticulously designed to maximize its radiative efficiency. It features width $W_{\mathrm{PA}}$ of 120 μm and length $L_{\mathrm{PA}}$ 64.1 μm. An imperative feature of the patch antenna is the simplistic control of the impedance to match the FET feed structure. Strategic perforations in the patch antenna near the feed and precise choice of permittivity values for the substrate and feed are adopted to optimize both radiation efficiency and impedance matching. The depth of the patch substrate, feed, and FET substrate are consistent at *l* = 4.95 μm. This depth allows the EM waves generated from the instability and reflected from the bottom metallic layer the ability to constructively interfere in the FET, and should allow efficient radiation in the terahertz regime for the microstrip patch antenna. The source and drain contacts are not required to extend to the bottom of the device; rather, only their connection to the graphene layer is pivotal. Consequently, the source and the bottom of the feed, both grounded, can be interconnected, effectively confining electromagnetic waves within the FET as they are positioned externally. Given that the drain, located within the device between the FET and the feed, is not grounded and only needs to establish contact with the graphene layer to supply the bias current, flexibility is possible in the depth of the drain contact to prevent a short-circuit. Our comprehensive simulations, which include sweeping the depth (*l*) of the FET and the resonant frequency of DS instability, reveal constructive interference on the graphene layer over a wide range of depths and resonant frequencies. This empirical evidence indicates that the chosen depth of *l* = 4.95 μm represents an optimal value for our targeted resonant frequency. Importantly, these findings suggest that the constructive interferences crucial for the device’s operation are not strictly contingent on a specific depth or resonant frequency, enhancing the adaptability and potential utility of our proposed graphene-based FET.

**Figure 2:**
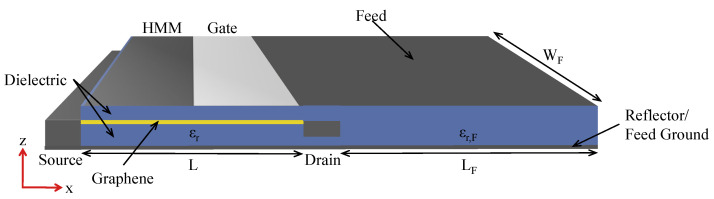
Schematic of the FET-feed system with the FET on the left and the attached feed on the right, to be fed into the patch antenna. A bottom reflector (extending across the bottom of the FET and feed) ensures maximum power is transferred to the gate with minimal leakage, and doubles as the ground plane for the feed.

**Figure 3:**
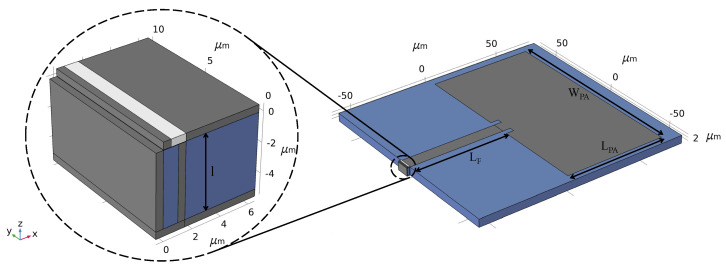
Schematic of the FET feed patch antenna system modeled in COMSOL. The inset shows the FET (**left** of the purple boundary) with the attached feed (**right** of the purple boundary), applied to the feed port of the antenna. The current is carried from the FET to the antenna through the feed in the x-direction.

The feed structure, seamlessly integrated with the antenna, is strategically situated to the right of the FET’s drain contact, where the gate that carries the current continues to the feed top contact (shown in Figure 2). It features dimensions of 60.5 μm in length ($L_{F}$) and 8 μm in width ($W_{F}$). The gate, contacts, and bottom reflector of the FET effectively contain the electromagnetic radiation within the enclosed region. This containment augments the coupling efficiency of plasmonic waves in graphene with the gate. Concurrently, the metallic bottom layer of the feed structure acts as the grounding plane for the feed and antenna, establishing a comprehensive electromagnetic circuitry. Figure 3 provides an overview of the entire setup encompassing the FET, feed, and microstrip patch antenna, with a detailed zoom-in on the FET structure.

## 3. Simulation Platform

In this section, we explain in detail the simulation platform utilized to comprehensively investigate the performance of the proposed microstrip patch antenna with an integrated graphene-based FET. The simulation methodology involves a dual-stage approach: first, we model the FET and feed system using MATLAB, where hydrodynamic equations for electron transport in graphene and Finite-Difference Time-Domain (FDTD) techniques for EM field modeling are employed, derived in [15,16]. The dimensions and complexity of the FET make MATLAB a suitable choice for the transient response of the system. Subsequently, we employ COMSOL Multiphysics to simulate the microstrip patch antenna. The larger dimensions of the patch antenna make COMSOL a suitable choice for the radiative characteristics of the system. Our previous attempts showed a transient response of the FET too long for the iterative solver to be modeled in COMSOL, and antenna dimensions too large for the FDTD solver in MATLAB. However, the FET is modeled in COMSOL without DS instability transient response to ensure impedance matching and to minimize reflections. This dual-stage simulation strategy enables us to address the inherent complexities of the device.

### 3.1. MATLAB-Based Simulation of the FET and Feed System

The FET and feed system are primarily modeled in MATLAB, where we employ hydrodynamic equations for electron transport in graphene. These equations encompass critical parameters such as electron density (*n*), electron current density (*j*), and drift velocity (*v*), which are interconnected through relationship j=nv. The electron density ($n_{0}$) is initialized with a depleted region following the Fermi function dependent on the applied gate voltage, where the doped graphene is considered to take the equilibrium density of $n_{0}$ = $6\times 10^{12}$ ${cm}^{-2}$ and the gated region is depleted to $n_{0}$ = $2\times 10^{12}$ ${cm}^{-2}$. At these densities, $E_{F}\gg T$, and the graphene channel is far from the Dirac point. The electron density (n(x,t)) is updated in the iterative loop using the continuity equation,

(2) $$\frac{\partial n}{\partial t}+\frac{\partial j}{\partial x}=0.$$

We utilize the Euler equation augmented with a scattering term to model the electron current density (j(x,t)). The Euler equation for massless 2D Dirac fermions to describe the electron dynamics in the DC current-biased graphene layer has the form

(3) $$\begin{array}{cc} \; & \left(2-\beta^{2}\right)\frac{\partial j}{\partial t}+2\beta v_{F}\frac{\partial j}{\partial x}+\left(1-2\beta^{2}\right)v_{F}^{2}\frac{\partial n}{\partial x}+\frac{2v_{F}{\left(1-\beta^{2}\right)}^{5/4}}{\sqrt{\pi }\hbar }\sqrt{n}eE_{x}^{ind}-\\ & \left(1-2\beta^{2}\right){\left(\frac{1-\beta^{2}}{1-\beta_{0}^{2}}\right)}^{5/4}v_{F}^{2}\sqrt{\frac{n}{n_{0}}}\frac{dn_{0}}{dx}+\frac{2\left(j-\beta_{0}v_{F}n\right){\left(1-\beta^{2}\right)}^{5/4}}{\tau }\sqrt{\frac{n_{0}}{n}}=0. \end{array}$$

Here, $\beta =\frac{v}{v_{F}}$, $n_{0}$ is the equilibrium electron density, $v_{0}$ = $2.8\times 10^{5}$ ${ms}^{-1}$ is the drift velocity due to stationary electron flow $j_{0}=n_{0}v_{0}=const$ in the channel, τ = 2.3 ps is the electron momentum relaxation time assumed in our model, $v_{F}=1.5\times 10^{6}\;{ms}^{-1}$ is the electron Fermi velocity in graphene, and −e is the electron charge. The self-consistent electric field $E_{x}$ is induced by the charged fluctuations in the electron system. For electron channels with non-uniform equilibrium electron density $n_{0}\left(x\right)$ considered in this paper, electric field $E_{x}$ includes a static built-in electric field $E_{0x}$ producing non-uniform equilibrium electron density distribution. Total field $E_{x}$ is split in Equation (3) as $E_{x}=E_{0x}+E_{x}^{ind}$, where $E_{x}^{ind}$ is the induced electric field. A small “kick” is applied to the initial current density for the onset of plasmonic reflections in the channel. These equations form the basis of our iterative solver, facilitating the study of charge transport in graphene within the FET.

To couple the EM field interactions, we employ the FDTD method to solve Maxwell’s equations. Specifically, we solve for the current density ($J=-e\left(j-j_{0}\right)\delta \left(z\right)\hat{x}$) using the hydrodynamic solver and subsequently input this current density into the FDTD-based EM solver to compute the electric field component ($E_{x}$),

(4) $$\begin{array}{cc} \nabla \times E^{ind} & =-\mu_{0}\frac{\partial H^{ind}}{\partial t},\\ \nabla \times H^{ind} & =J+\epsilon \epsilon_{0}\frac{\partial E^{ind}}{\partial t}, \end{array}$$

where $E^{ind}=E_{x}^{ind}\hat{x}+E_{z}^{ind}\hat{z}$ and $H^{ind}=H_{y}^{ind}\hat{y}$ are the electric and magnetic components of the EM field induced by the electric current J in the channel. This electric field distribution ($E_{x}$) is then fed back into the hydrodynamic solver, creating a closed-loop self-consistently solved iterative process that ensures a comprehensive analysis of the collective plasma excitations in the 2D graphene channel of the FET and the accompanying electromagnetic radiation generated in the instability regime. The resulting steady-state current at the feed-end of the HMM gate is recorded and subsequently transferred to COMSOL Multiphysics as the antenna feed input.

### 3.2. COMSOL-Based Simulation of the Microstrip Patch Antenna

In the second stage of our simulation approach, we employ COMSOL Multiphysics to model the microstrip patch antenna integrated with the FET. Due to the large dimensions of the antenna structure, a full electromagnetic simulation using the FDTD in MATLAB becomes impractical. Instead, we opt for the transient interface simulation technique using the steady-state current extracted from MATLAB.

In this COMSOL simulation, we inject the recorded current from the MATLAB simulation into the feed port of the microstrip patch antenna. The steady-state response is carefully observed over a short time frame, allowing us the ability to analyze the antenna’s radiation characteristics. Importantly, the impedance matching is pre-established, mitigating the need to account for reflections and simplifying the analysis. This is achieved first by calculating the impedance of the FET and the feed in MATLAB, as well as the feed and microstrip patch antenna in COMSOL, and optimizing the parameterized dimensions and materials to match the impedances. This ensures that there are minimal reflections to the FET and confirms the onset of DS instability is not hindered by the attached feed. Following this, the impedance match is ensured by attaching an inactive FET to the antenna-feed system in COMSOL and minimizing the reflections to the FET-feed port at the resonant frequency.

This dual-stage simulation strategy should provide a comprehensive insight into the device’s operation. While MATLAB is utilized to investigate the intricate charge transport within the FET and its coupling with the EM field, COMSOL is employed to scrutinize the antenna’s radiative performance, especially when dealing with dimensions beyond the capabilities of the MATLAB FDTD model and transient response times that exceed the feasibility of iterative solvers in COMSOL.

## 4. Results

In this section, we present the results of our comprehensive simulation and analysis of the microstrip patch antenna integrated with graphene-based FET. These results should shed light on the device’s charge transport dynamics, impedance matching, radiative characteristics, and key operating frequency.

**Figure 4:**
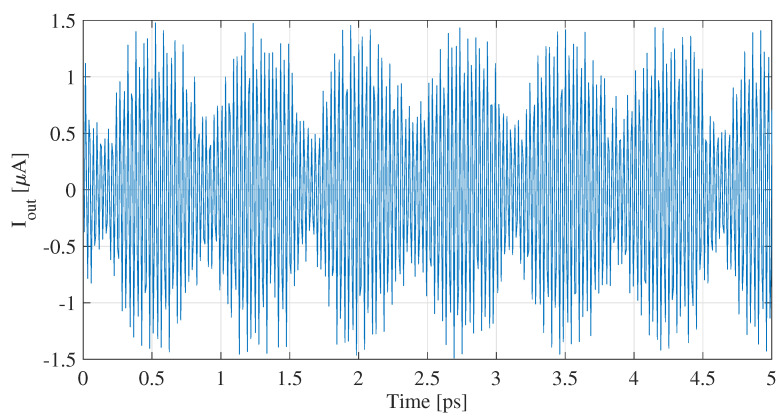
Time-domain plot of current recorded on the FET gate.

We begin with a detailed examination of the time-domain plot of the recorded current at the end of the FET gate, where the HMM meets the feed, as depicted in Figure 4. This plot provides insights into the coupling efficiency of the plasmons from the graphene layer to the gate and HMM. With minimal leakage confirmed from the simulations and a measured current in the μA range, a high coupling efficiency is ascertained. This signal is applied to the port of the feed in COMSOL.

**Figure 5:**
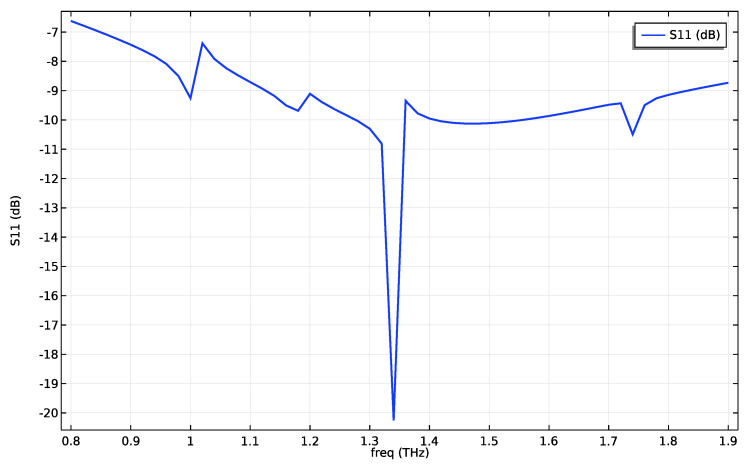
The S11 curve of the FET antenna showing minimal reflections at 1.34672 THz.

Next, we turn our attention to the S11 curve of the FET antenna system, illustrated in Figure 5. The S11 curve characterizes the reflection coefficient and, significantly, reveals the impedance matching capabilities of the antenna system. Notably, the S11 curve showcases minimal reflections at a frequency of 1.34672 THz, the resonant frequency of the current received from the FET, demonstrating optimal impedance matching and minimal power loss due to reflections. The plot shows a bandwidth of 80 GHz under the −10 dB threshold, which from ref. [17] corresponds to a gate voltage change of 0.6–0.9 V due to the nonlinear dependence of gate voltage and fundamental frequency. This variance in voltage has a minimal impact on the impedance of the FET at the feed port, as our simulations have shown changes within 1 Ω real impedance and 0.4 Ω imaginary impedance.

**Figure 6:**
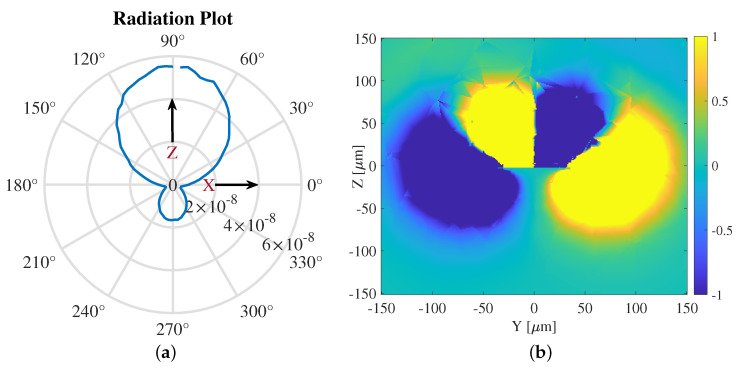
(**a**) Radiation pattern of the FET antenna. (**b**) EM field distribution of the FET antenna system, with the microstrip patch antenna centered at x=0μm and y=0μm.

Regarding the radiative characteristics, we present the radiation pattern of the FET antenna in Figure 6a and the 2D EM field distribution via a 2D time-averaged Poynting vector in Figure 6b with the *x*-axis fixed at the center of the patch antenna. The 2D representation of the radiation pattern allows us the possibility to assess the antenna’s directivity, beamwidth, and angular distribution of radiated power. The simulations yielded a directivity of *D* = 52.7 dBi, with a radiative power of 2.2 μW. For comparison, the detached asymmetric FET in [16] without a bottom reflector or HMM provides 45 nW of radiative power. This large enhancement comes from the efficient coupling of the plasma waves to the gate, and the confinement of terahertz radiation within the FET to efficiently transfer to the feed and antenna. The FET antenna field plot, obtained from COMSOL and transferred to MATLAB, shows the field characteristics of the system in the transient interface with the current extracted from the FET. The microstrip patch antenna is centered on the *x*-axis and the top of the antenna is aligned with z= 0.

**Figure 7:**
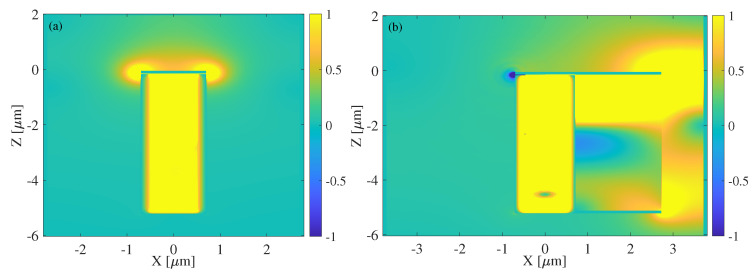
(**a**) EM field distribution of the FET, including the metallic/HMM gate and bottom reflector, showing EM radiation confined in the cavity. (**b**) EM field distribution of the FET-feed system, demonstrating efficient power flow from the FET to the feed.

The 2D EM field distribution is analyzed using a time-averaged Poynting vector, first of the FET in Figure 7a then of the FET-feed configuration in Figure 7b. The FET field plot, obtained from MATLAB with the device containing the metallic gate on the source side, HMM gate on the drain side, and the bottom reflector, confirms minimal leakage from the stand-alone device to ensure maximum confinement within the FET. A small amount of radiative leakage is found at the top, where the boundary conditions required for the onset of DS instability are sensitive, thus a small space between the gate and source/drain contacts is necessary. The FET with the feed attached to the right of the drain shown in Figure 7b confirms the containment of the EM radiation within the FET for maximum power transfer to the feed. The small leakage is still observed on the source side of the FET; however, the current and EM power are visibly transferred mostly to the gate–feed extension.

The combined examination of these results offers a comprehensive understanding of the FET antenna system, its resonance behavior, and its ability to efficiently radiate terahertz signals at the resonant frequency.

## 5. Conclusions

In conclusion, our investigation of the microstrip patch antenna integrated with a graphene-based FET has revealed promising prospects for active terahertz signal radiation. The FET’s charge transport dynamics and electrodynamics have been explored, leading to insights into transient behavior and power efficiency. Notably, our study identified a resonant frequency of 1.34672 THz, where the FET antenna system exhibited optimal impedance matching and minimal reflections, making it a robust candidate for terahertz signal generation.

The radiation pattern analysis of the FET antenna showcased its favorable directional properties, highlighting its potential for applications in terahertz communication, sensing, and imaging. The device’s high degree of tunability for impedance matching further underscores its versatility. Overall, this research provides valuable insights into the design and performance of terahertz radiation systems, offering a promising path for advancements in this critical frequency range.

## Data Availability

Data are contained within the article.
